# Human Infection with Influenza Virus A(H10N8) from Live Poultry Markets, China, 2014

**DOI:** 10.3201/eid2012.140911

**Published:** 2014-12

**Authors:** Tao Zhang, Yuhai Bi, Huaiyu Tian, Xiaowen Li, Di Liu, Ying Wu, Tao Jin, Yong Wang, Quanjiao Chen, Ze Chen, Jianyu Chang, George F. Gao, Bing Xu

**Affiliations:** Ministry of Education Key Laboratory for Earth System Modelling, Center for Earth System Science and School of Environment, Tsinghua University, Beijing, China (T. Zhang, Y. Wang, B. Xu);; Chinese Academy of Sciences Institute of Microbiology, Beijing (Y. Bi, D. Liu, Y. Wu, G.F. Gao);; Beijing Normal University, Beijing (H. Tian, X. Li, B. Xu); BGI-Shenzhen, Shenzhen, China (T. Jin);; Wuhan Institute of Virology, Chinese Academy of Sciences, Wuhan, China (Q. Chen, Z. Chen);; China Agricultural University, Beijing (J. Chang);; Chinese Center for Disease Control and Prevention, Beijing (G.F. Gao)

**Keywords:** avian influenza, H10N8, live poultry markets, phylogenetic analysis, human infection, influenza, viruses, transmission, China

## Abstract

Human infection with avian influenza virus A(H10N8) was initially reported in China in December 2013. We characterized H10N8 strains from a human patient and from poultry in live markets that infected persons had visited. Results of genome sequencing and virus characterization suggest that the virus strains that infected humans originated from these markets.

Avian influenza virus (AIV) is classified into 16 subtypes on the basis of hemagglutinin (HA) and 9 subtypes on the basis of neuraminidase (NA); additional bat-derived influenza-like genomes, H17N10 and H18N11, have recently been reported (*1*). Birds can be infected with AIV through direct contact with infected hosts or through contact with contaminated surfaces or materials, including water and food. In China, H10N8 virus was isolated from the environment of Dongting Lake in Hunan Province in 2007 (*2*) and from a duck in a live poultry market (LPM) in Guangdong Province in 2012 (*3*). This AIV was not then known to directly infect humans or other mammals. 

In December 2013, H10N8 virus infection in a person was reported in Nanchang, Jiangxi Province, China (*4*); 2 more human cases followed. The initial reported case was in a 73-year-old woman who visited a local LPM 4 days before the onset of her illness (*4*). Because genetic information on AIV is essential for understanding of the biology of these viruses, their spread among avian species, and their potential transmission to humans, in January 2014, we conducted surveillance of several LPMs in Nanchang, including those visited by the 3 reported case-patients, to determine the source of these infections.

## The Study

During January 2014, we collected 226 pairs of cloacal and oropharyngeal swab specimens from apparently healthy poultry in several LPMs in Nanchang, China. The samples were stored in viral medium at 4°C until they were transported to the laboratory and then stored at −80°C until analysis. Virus material was injected into 10-day-old specific pathogen free embryonated chicken eggs; we then genetically analyzed all HA-positive samples. Viral RNA from allantoic fluid was extracted with the RNeasy Mini Kit (QIAGEN, Hilden, Germany), and cDNAs were synthesized from the viral RNA by reverse transcription PCR by using a SuperScript First-Strand Synthesis System for RT-PCR Kit (Invitrogen, Carlsbad, CA, USA). All gene segments were amplified by using a Phusion High-Fidelity PCR Kit (New England Biolabs, Ipswich, MA, USA). The PCR products were sequenced and the sequences were edited and aligned by using BioEdit software (http://www.mbio.ncsu.edu/bioedit/bioedit.html).

**Figure 1:**
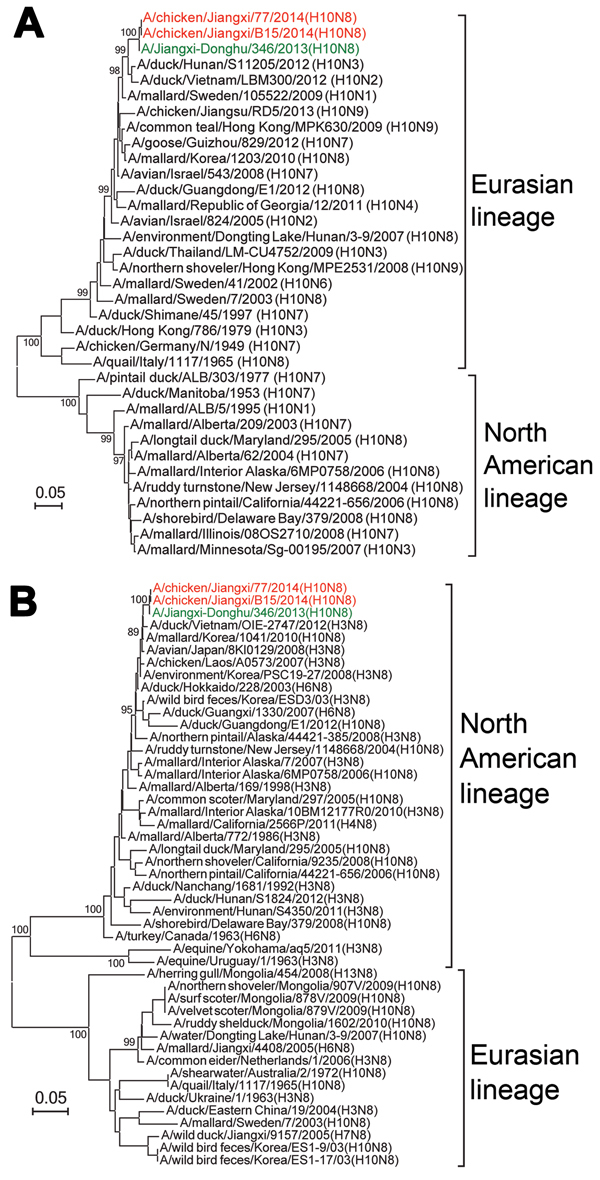
Phylogenetic trees of hemagglutinin (A) and neuraminidase (B) gene segments of influenza virus A(H10N8) isolates from Jiangxi Province, China, 2013–2014, compared with other closely related influenza viruses. Red indicates the novel H10N8 isolates A/chicken/Jiangxi/77/2013 (H10N8) and A/chicken/Jiangxi/B15/2014 (H10N8) that were identified in this study from poultry from live poultry markets; green indicates the human-source H10N8 virus isolate A/Jiangxi/346/2014 (H10N8). Scale bars indicate nucleotide substitutions per site.

**Figure 2:**
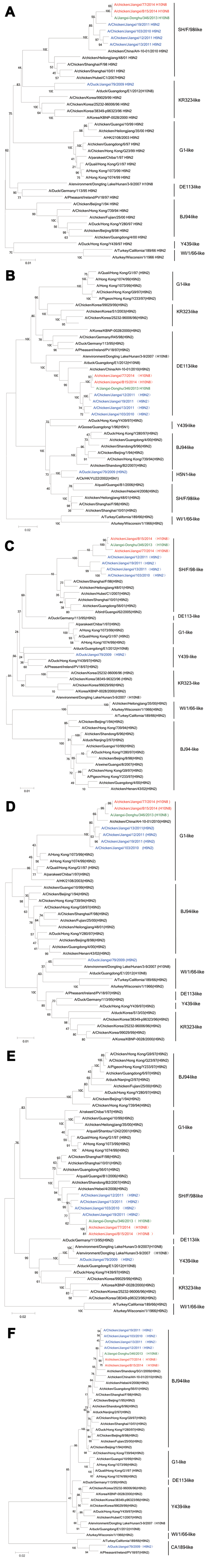
Phylogenetic trees of the internal genes of influenza virus A(H10N8) isolates from Jiangxi Province, China, 2013–2014, compared with other closely related influenza viruses. A) Polymerase basic 1; B) polymerase basic 2; C) nucleoprotein; D) matrix; E) polymerase acidic; F) nonstructural. Red indicates the novel H10N8 isolates A/chicken/Jiangxi/77/2013 (H10N8) and A/chicken/Jiangxi/B15/2014 (H10N8) that were identified in this study from poultry from live poultry markets; green indicates the human-source H10N8 virus isolate A/Jiangxi/346/2014 (H10N8); blue indicates the 5 H9N2 virus isolates used for comparison, A/environment/Jiangxi/00449/2013 (H9N2), A/chicken/Jiangxi/103/2010 (H9N2), A/chicken/Jiangxi/12/2011(H9N2), A/chicken/Jiangxi/19/2011 (H9N2), and A/chicken/Jiangxi/13/2011 (H9N2). Scale bars indicate nucleotide substitutions per site.

Two H10N8 viruses, A/chicken/Jiangxi/77/2014 (JX77) and A/chicken/Jiangxi/B15/2014 (JXB15), were isolated from the 226 pairs of swab specimens from healthy chickens. JXB15 and JX77 grew well in embryonated eggs and caused the specific pathogen free chicken embryos to die 48–72 h after inoculation; hemagglutination titers were 2^6–7^. Genomic analysis showed that these isolates had sequence identity of >99% in each of the 8 genes tested with human isolate A/Jiangxi-Donghu/346/2013 (H10N8) (JX346), which was derived from the case-patient who became ill in December 2013. This finding indicates that the H10N8 virus that infected humans might have been derived from local poultry or the environments of LPMs. All 8 genes of JXB15 and JX77 were adjacent to the corresponding genes of JX346 on phylogenetic trees (Figures 1, 2). JXB15 and JX77 also shared high nucleotide sequence identity (99.3%–99.9%) with JX346 (Table 1). Phylogenetic analysis showed that the HA genes of JXB15 and JX77 belong to the Eurasian avian lineage, whereas the NA genes belong to the North American avian lineage (Figure 1). When compared with JX346, the avian isolates JXB15 and JX77 contained nucleotide differences resulting in only 5 amino acid substitutions in the HA protein: Met80Tyr, Asn116Asp, Thr188Ile, Lys415Met, and Phe536Val (Table 2). All 3 isolates shared 100% identity in their NA amino acid sequences. 

**Table 1:** Nucleotide identity of influenza A(H10N8) and A(H9N2) viruses isolated in Jiangxi Province, China, during 2009–2014, compared with human isolate A/Jiangxi-Donghu/346/2013 (H10N8)*

Strain	Collection date	Nucleotide identity, %							
HA	NA	PB2	PB1	PA	NP	M	NS
A/Jiangxi/346/2013 (H10N8)	2013 Dec								
A/chicken/Jiangxi/77/2014 (H10N8)	2014 Jan	99.5	99.8	99.5	99.9	99.9	99.3	99.4	99.9
A/chicken/Jiangxi/B15/2014 (H10N8)	2014 Jan	99.5	99.8	99.5	99.9	99.9	99.7	99.4	99.9
A/environment/Jiangxi/00449/2013 (H9N2)	2013 Feb	NT	NT	98.6	96.4	98.6	99.5	99.4	98.8
A/chicken/Jiangxi/103/2010 (H9N2)	2010 Dec	NT	NT	97.8	96.3	98.1	96.3	98.6	98.6
A/chicken/Jiangxi/12/2011 (H9N2)	2011 Jan	NT	NT	97.2	97.3	97.3	97.7	98.8	98.8
A/chicken/Jiangxi/13/2011 (H9N2)	2011 Jan	NT	NT	97.4	97.4	97.6	95.9	98.6	98.5
A/chicken/Jiangxi/19/2011 (H9N2)	2011 Jan	NT	NT	97.1	98.1	97.6	96.1	98.9	98.9
A/duck/Jiangxi/79/2009 (H9N2)	2009 Sep	NT	NT	87.0	90.0	89.8	91.1	90.7	69.4

*Isolates were from mixed cloacal and oropharyngeal swab specimens collected in Nachang city, Jiangxi Province. Nucleotide identity was calculated by using DNAStar software (http://www.dnastar.com) from the complete coding sequence of each gene segment. HA, hemagglutinin; M, matrix protein; NA, neuraminidase; NP, nucleoprotein; NS, nonstructural protein; NT, not tested; PA, polymerase acidic protein; PB, polymerase basic protein.

**Table 2:** Molecular characteristics of human influenza A(H10N8) isolate A/Jiangxi-Donghu/346/2013 (H10N8), H10N8 isolates collected from live poultry markets, and other H10N8 and H9N2 viruses isolated in China, 2007–2014*

Virus strain	HA			NA					PB2						PB1			M1			M2		NS1
	Connecting peptide	RBS		Deletion	119	274	292		89	357	591	627	701		473	598		30	215		31		42
				69–73	Glu	His	Arg		Val	His	Gln	Glu	Asp		Val	Leu		Asp	Ala		Asn		Ser
A/Jiangxi-Donghu/346/2013 (H10N8)	KLIGR↓GL	QSG		–	+	+	+		+	+	+	Lys	+		+	+		+	+		+		+
A/chicken/Jiangxi/B15/2014	KLIGR↓GL	QSG		–	+	+	+		+	+	+	+	+		+	+		+	+		+		+
A/chicken/Jiangxi/77/2014	KLIGR↓GL	QSG		–	+	+	+		+	+	+	+	+		+	+		+	+		+		+
A/duck/Guangdong/E1/ 2012 (H10N8)	KLVGR↓GL	QSG		–	+	+	+		+	+	+	+	+		+	+		+	+		Ser		+
A/environment/Dongting Lake/Hunan/3-9/2007 (H10N8)	KFIGR↓GL	QSG		–	+	+	+		+	+	+	Lys	+		+	+		+	+		Ser		+
A/chicken/Jiangxi/12/2011 (H9N2)									+	+	+	+	+		+	+		+	+		+		+
A/chicken/Jiangxi/13/2011 (H9N2)									+	+	+	+	+		+	+		+	+		+		+
A/chicken/Jiangxi/19/2011 (H9N2)									+	+	+	+	+		+	+		+	+		+		+
A/duck/Jiangxi/79/2009 (H9N2)									+	+	+	+	+		+	+		+	+		Ser		Ala
A/chicken/Jiangxi/103/2010 (H9N2)									+	+	+	+	+		+	+		+	+		+		+

*Ala, alanine; Asn, asparagine; Asp, aspartic acid; Gln, glutarnine; Glu, glutamic acid; HA, hemagglutinin; His, histidine; Leu, leucine; Lys, lysine; M, matrix protein; NA, neuraminidase; NP, nucleoprotein; NS, nonstructural protein; PA, polymerase acidic protein; PB, polymerase basic protein; RBS, receptor binding sites; Ser, serine; Val, valine.

Phylogenetic trees constructed from the 6 internal genes (polymerase basic [PB] 1 and 2, polymerase acidic, nucleoprotein, matrix [M], and nonstructural [NS]) showed that JXB15, JX77, and JX346 clustered closely with H9N2 viruses isolated from Jiangxi in 2010 and 2011 (Figure 2). The internal genes of JXB15 and JX77 also showed high nucleotide sequence identity with those of 5 H9N2 virus isolates collected from local poultry environments during 2010–2013, with highest similarity to those of A/environment/Jiangxi/00449/2013 (H9N2) (96.4%–99.5%) (Table 1). These findings suggest that the 6 internal genes of these 3 H10N8 viruses originated from local H9N2 viruses that have been circulating for years. The 6 internal genes of JXB15 and JX77 shared 95.9%–98.9% identity with those of the H9N2 viruses from chickens but only 69.4%–91.1% with those of H9N2 viruses from ducks (Table 1). Phylogenetic analysis showed that the PB2 genes of JXB15, JX77, and JX346 belong to the DE113-like lineage; the PB1, polymerase, and nucleoprotein genes belong to the SH/F/98-like lineage; the M genes belong to the G1-like lineage; and the NS genes belong to the BJ94-like lineage (Figure 2).

Amino acid sites 591K, 627K, and 701N in the internal protein PB2 are key amino acids required for the mammalian adaptation of the virus (*5*). JXB15, JX77, and JX346 have 591Q and 701D in protein PB2; however, although the human JX346 virus has 627K in protein PB2, 627E is found in the poultry isolates. This substitution suggests that the mammalian-adaptation mutation to 627K might have occurred in the human body after infection. For all 3 isolates, amino acids 473V and 598L were also observed in the viral PB1 protein in mammalian cells and are assumed to enhance the replicative capacity of the virus (*6*). Deletions of the PDZ motif and 42Ser in the NS1 protein were observed; these changes have been shown to increase pathogenicity in mice (*7*). The M2 protein contained the Ser31Asn substitution, indicating resistance to adamantanes, as has been consistently observed in recent seasonal influenza virus isolates.

## Conclusions

Our results provide evidence that the novel avian influenza virus A(H10N8) that infected humans in Nanchang, Jiangxi Province, China, could have derived from strains circulating in LPMs. In the LPMs, the sale of freshly slaughtered poultry, live poultry transportation, and mixed trading of different domestic animals provide environments conducive to genome segment reassortment, gene mutation, and interspecies transmission of AIVs (*8*,*9*). Human-infecting H7N9 virus strains are believed to be directly related to those found in the live poultry traded in LPMs (*10*,*11*); closure of LPMs has been shown to partly control the spread of these infections (*8*). Moreover, serologic evidence recently confirmed the infection of dogs with an H10 subtype influenza virus in close proximity to LPMs in Guangdong Province (*12*). Other recent research has shown that the internal genes of the H5N1, H7N9, and H10N8 viruses are constantly reacquired from poultry H9N2 viruses (*9*,*13*,*14*). Taken together, these data suggest that LPMs act as gene sources, facilitating reassortment of AIV genome segments (*15*).

In summary, exposure to infected and/or virus-carrying poultry or to contaminated environments in LPMs and the emergence of mammal-adapted and drug-resistant viruses puts humans at high risk for infection with novel influenza viruses. Measures to improve poultry farming practices must be enforced, including strict biosecurity measures for the trade and transport of live birds, proper disposal of diseased and dead birds, and even closure of LPMs.
